# Advances in Mechanisms of Anaphylaxis in Wheat Allergy: Utility of Rodent Models

**DOI:** 10.3390/foods14050883

**Published:** 2025-03-05

**Authors:** Tamil Selvan Arul Arasan, Rick Jorgensen, Chris Van Antwerp, Perry K. W. Ng, Venu Gangur

**Affiliations:** 1 Food Allergy and Immunology Laboratory, Department of Food Science and Human Nutrition, Michigan State University, East Lansing, MI 48824, USA; arultami@msu.edu (T.S.A.A.); jorgen70@msu.edu (R.J.); vanant29@msu.edu (C.V.A.); 2 Cereal Science Laboratory, Department of Food Science and Human Nutrition, Michigan State University, East Lansing, MI 48823, USA; ngp@msu.edu

**Keywords:** wheat, wheat gluten, wheat non-gluten, anaphylaxis, animal models, IgE, exercise, genetics, environment

## Abstract

Wheat is a staple and nutritious food that is consumed globally. However, it is identified as a major allergenic food because of its capacity to trigger life-threatening systemic anaphylaxis. The specific mechanisms that underlie this systemic anaphylaxis in wheat allergy are incompletely understood. As a result, several rodent models have been developed to study anaphylaxis in wheat allergies. In this paper, we have conducted a comprehensive review of wheat-induced anaphylaxis using Google Scholar and PubMed databases with relevant keywords. The following objectives were addressed: (1) to determine the complexity of wheat-induced anaphylaxis; (2) to summarize the role of genetic susceptibility in wheat anaphylaxis; (3) to identify the environmental factors involved in the development of wheat anaphylaxis; (4) to map the current status of mechanisms involved in wheat anaphylaxis; (5) to identify the approaches, strengths, and limitations of rodent models of wheat anaphylaxis; and (6) to identify challenges and opportunities in this area of science. Our findings provide a comprehensive updated critical resource for the future research agenda in wheat allergy-associated anaphylaxis, particularly using rodent models as attractive pre-clinical tools.

## 1. Introduction

Wheat is one of the most prominent foods globally that has been in the human diet for a long period of time (at least 10,000 years) [1]. Although wheat serves as a staple food in different parts of the world, allergy caused by wheat proteins such as gluten is a growing concern among people in developed countries, as well as in some of the developing countries in Asia, such as China, Hong Kong, Singapore, Malaysia, and India [2]. Consequently, food safety regulations are enforced in many countries, including the USA, Canada, European Union countries, and Australia, to prevent and manage potential wheat allergic reactions [3,4,5,6,7]. Wheat is regulated as a major food allergen in these countries [8].

In the USA, between 0.4% and 4.3% of the population has wheat allergies [9,10,11]. There is ample evidence that the prevalence of food allergies in general and wheat allergies in particular has increased dramatically over the past three decades [12]. A major concern for public safety from wheat allergies is the potential of deadly reactions known as systemic anaphylaxes, where multiple vital organs (heart, lungs, brain), in addition to gut and skin, are involved in the disease elicitation [13]. However, the underlying deadly reactions of the mechanism are incompletely understood at present.

The general mechanism of food allergy development involves two phases: It begins with sensitization to the concerned food, where subjects induce IgE antibodies, followed by disease elicitation upon subsequent exposure to the concerned food. Before sensitization, there are no clinical reactions to the food. Therefore, sensitization is a required factor for the subsequent expression of clinical disease symptoms. When allergic reactions are limited to the gut (for example, diarrhea, vomiting), it is not considered as systemic anaphylaxis [14,15]. However, when symptoms expand to vital organs (heart, lungs, brain) in addition to the gut, the reaction is regarded as systemic anaphylaxis that is potentially fatal [16,17,18]. There has been marked progress recently in the classification of anaphylaxis as reported in recent papers [19,20,21]. Turner et al. (2024) proposed a modification of the previous WAO grading system for systemic anaphylactic reactions, which aligns closely with version 3.0 of the Consortium for Food Allergy Research (COFAR) grading scale [20,21]. According to this proposal, allergic reactions are classified into five grades based on objective clinical parameters of disease manifestation. Reactions in grades 1 and 2 involve skin, gut, or other systems with mild symptoms. Grades 3 to 5 involve additional reactions affecting the cardiovascular and neurological systems. So, the definition of anaphylaxis per this proposal requires clinical reactions from grade 3 to grade 5. The present study was conducted to develop a comprehensive review on the subject and identify specific topics for future research.

In this study, the following objectives were addressed: (1) to determine the complexity of wheat anaphylaxis; (2) to summarize the role of genetic susceptibility in wheat anaphylaxis; (3) to identify the environmental factors involved in the development of wheat anaphylaxis; (4) to map the current status of mechanisms involved in wheat anaphylaxis; (5) to identify the approaches, strengths, and limitations of rodent models of wheat anaphylaxis; and (6) to identify challenges and opportunities to advance science in wheat anaphylaxis. In order to address the above mentioned objectives, we used keywords such as “wheat”, “anaphylaxis”, “animal model”, “human”, and “gluten” in various combinations in PubMed and Google Scholar for this research. The output was evaluated, and objective-relevant information was obtained. The results were synthesized into concepts, figures, and tables as presented.

This review has identified the complexity associated with wheat-induced anaphylaxis in humans as well as rodent models. We have identified specific gaps and opportunities for future research to enable the development of improved methods for the prevention, treatment, and management of wheat-induced anaphylaxis. Our findings provide a comprehensive updated critical resource for developing the future research agenda in wheat allergy-associated anaphylaxis using rodent models as very attractive pre-clinical tools.

## 2. Complexity of Anaphylaxis in Wheat Allergy: Current Status

Anaphylaxis is clinically defined as a potentially deadly reaction of the immune system to certain environmental agents including insect stings, drugs, radiological contrast media, and allergenic foods [22]. In the USA, the following nine foods are regulated for their capacity to trigger such deadly reactions: wheat, egg, milk, soy, peanut, tree nut, fish, shellfish, and sesame [7]. The scope of this article is limited to wheat allergy-associated anaphylaxis.

### 2.1. Overall Classification of Types of Anaphylaxis in Wheat Allergy

Anaphylaxis is triggered by different types of agents, and the clinical characteristics leading to the deadly condition are largely similar. However, based on the mechanisms involved, it is classified into three major groups: (i) IgE antibody-mediated anaphylaxis; (ii) non-IgE-mediated anaphylaxis; and (iii) idiopathic anaphylaxis with unknown mechanisms [23]. It is noteworthy that all reported systemic anaphylaxes in wheat allergy belong to the first group since they are all mediated by the wheat-specific IgE antibodies [24]. Furthermore, based on whether cofactors such as exercise, etc., are required in addition to wheat consumption, anaphylaxis in wheat allergy is classified into two groups: (i) classical systemic anaphylaxis upon wheat exposure and (ii) cofactor-dependent systemic anaphylaxis in wheat allergy. In the first group, wheat-sensitized patients react within minutes upon re-exposure to wheat. In the second group, wheat-sensitized subjects react to wheat only when they are exposed to other factors simultaneously (alcohol, infection) or within a short time (exercise) after exposure to wheat. The latter condition is known as wheat-dependent exercise-induced anaphylaxis (WDEIA) [25].

### 2.2. Role of Host Genetic Susceptibility in the Development of Wheat Allergy and Anaphylaxis

Wheat is a staple food consumed by billions of people around the world. However, only a small but significant number of individuals who are exposed to wheat allergens develop a wheat allergy. It is therefore clear that the genetic susceptibility to developing a wheat allergy is different among different people. There are eight studies reporting specific genetic factors associated with wheat allergies (Table 1). These studies and their significance are reviewed below.

The first study to identify the genetic risk of developing a wheat allergy was conducted by Cho et al. (2011) in a South Korean adult population. They investigated the relationship between polymorphism at the TLR4 locus and the risk of developing respiratory wheat allergy [26]. They found that the homozygous variant of two TLR4 alleles was associated with significantly reduced risk of developing respiratory wheat allergy. TLR4 encodes for the toll-like receptor, which is an innate receptor with a critical function in immune responses to both microorganisms and allergens [27]. It serves as a receptor for endotoxin lipopolysaccharide (LPS) present in the outer membrane of gram-negative bacteria according to hygiene hypothesis. Exposure to LPS may be protective against the development of allergic diseases [28]. Therefore, results from this study support the hygiene hypothesis related to respiratory wheat allergy development.

**Table 1:** Genetic susceptibility to developing wheat allergy in humans: current evidence.

Genetic Factors	Evidence from the Study	References
TLR4	Lower risk of respiratory wheat allergy was associated with *TLR4* polymorphism as follows: homozygotes for the −2027 G and −1608 C alleles (*n* = 381, adults, South Korean bakers study).	[26]
IL-4	Single nucleotide polymorphism at the *IL-4* locus (IL-4-C590T) was associated with WDEIA; Chinese study, *n* = 51, Age 5–77 years.	[29]
IL-4R	Single nucleotide polymorphism at *IL-4R* alpha locus (*IL-4RA* A1727G) was not associated with WDEIA; Chinese study, *n* = 51, Age 5–77 years.	[29]
	Increased positive skin-prick test to wheat flour in bakery workers (*n* = 373, South Korean study, adults) was associated with polymorphic variant of *IL-4Rα* (Ile375Val and Gln576Arg polymorphisms).	[30]
*Filaggrin gene*	A patient (woman age 51) had developed WDEIA upon using detergents containing HWP (Glupearl); however, she had no mutation in filaggrin gene that had been implicated for skin sensitization in Japanese subjects.	[31]
	In a Japanese family, a mother–daughter pair with the same filaggrin loss-of-function mutation developed WDEIA; the daughter was compound heterozygous for c.441_442delAG (p.Gly149Glufs*4) and c.5368C > T (p.Gln1790Ter), and the mother was heterozygous for c.441_442delAG.	[32]
	In a Denmark population (*n* = 7931, age: 18–69), filaggrin gene loss-of-function mutation was associated with self-reported food allergy, including wheat allergy, but not oral allergy syndrome (OR for wheat allergy 3.59; 95% CI 1.61–8.02).	[33]
HLA-class II variants	HLA class II DPB1*02:01:02 allele was associated with increased risk of WDEIA; Japanese population study, *n* = 77, adults.	[34]
	HLA class II (HLA DQ) locus on chromosome 6p21 was associated with wheat allergy (skin, eye, airways symptoms when used soap containing hydrolyzed wheat protein and/or skin, eye, airways, gut, and shock symptoms upon eating wheat products/SPT, IgE, basophil activation positive); Japanese population study, *n* = 452, adults.	[35]
RBFOX1	RBFOX1 locus on chromosome 16p 13 locus was associated with wheat allergy; same population as above.	[35]
IL-18	Increased risk of WDEIA was associated with *IL-18* gene locus (haplotype AGG); (*n* = 130, Han Chinese study, adults).	[36]
	Increased risk of sensitization to wheat among South Korean bakery workers was associated with IL-18 polymorphism (373 adults; South Korean study Genotype 137G/C (GC or CC) and haplotype *ht3* [ACC].	[37]
MBL	Higher levels of blood MBL are associated with increased risk of baker’s asthma in Korean population (*n* = 273); MBL levels were associated in the *MBL2* gene haplotypes.	[38]
Family genetics aggregation study	IgE-mediated food allergy trait (including wheat allergy) was associated with estimated heritability of 0.15–0.35; American nuclear family study (*n* = 581).	[39]

It is well established that allergic diseases in general, including wheat allergies, are mediated by the T helper 2 immune response to allergens that helps allergen-specific B cells to produce IgE antibodies [40]. Cytokine IL-4 is the prototypic T helper 2-derived cytokine implicated in IgE class switching [41,42]. Cai et al. studied genetic polymorphism at the IL-4 and IL-4R alpha locus in relation to susceptibility to WDEIA in Chinese subjects (children and adults) (Table 1). They found a significant association of SNP at the IL-4 locus but not the IL-4R alpha locus with WDEIA (Table 1). In contrast, Hur et al. (2013) reported the association of IL-4 receptor alpha locus with sensitization to wheat among South Korean bakery workers [30] (Table 1). These results suggest that genetic control at the IL-4 and IL-4R plays (or may play) a critical role in the development of wheat allergy.

Filaggrin is an important protein required for skin barrier function [43]. There are several studies implicating that mutation of the filaggrin gene results in the development of food allergies, such as peanut allergy [33]. There are, however, only two studies reporting the relationship between filaggrin loss-of-function mutation in three Japanese subjects with WDEIA condition (Table 1). In the first study, researchers found that a Japanese woman had developed WDEIA after skin exposure to a facial soap containing Glupearl, which is a hydrolyzed wheat gluten. However, this person did not carry a loss-of-function mutation in the filaggrin gene, suggesting that such mutation is not necessary to develop WDEIA upon skin exposure to wheat allergens. In the second study, the researchers found that a mother–daughter pair both carrying loss-of-function mutation polymorphism in the filaggrin gene developed WDEIA. Together, these studies suggest that WDEIA development does not necessarily require filaggrin gene mutation.

Linneberg et al. (2013) studied the role of filaggrin gene loss-of-function mutation in self-reported food allergies, including wheat allergy. The study population was adult Danish subjects [33]. Interestingly, individuals with this mutation exhibited an increased tendency to have food allergies in general, with 3.59 times more risk of developing wheat allergies (Table 1).

Noguchi et al. (2019) studied genetic polymorphism in HLA class II variants (HLA DQ) and RBFOX1 in a Japanese cohort of wheat-allergic subjects with symptoms of airways, skin, gut allergy, and anaphylaxis [35]. They found a positive association for both genes. HLA class II is critical for allergen presentation by antigen-presenting cells. There is a report associating the RBFOX1 gene with the development of pediatric food allergy in general [44].

Fukunaga et al. (2021) studied the genetic contribution of HLA class II to increasing the risk of developing WDEIA in Japanese adults [34] (Table 1). They identified a specific HLA class II DPB1 allele associated with increased risk of developing WDEIA.

Interleukin 18 plays a key role in immune responses and inflammation [45]. There are two studies investigating IL-18 polymorphisms in wheat allergy. The first study reported increased risk of developing WDEIA among Han Chinese adults associated with IL-18 Haplotype AGG. Another study examined the role of IL-18 genetics in developing wheat allergy among South Korean bakers. They identified two genetic variants linked with wheat allergy [37].

Kim et al. (2017) reported a relationship between the innate immune system marker, mannose-binding lectin (MBL) levels, gene polymorphisms, and baker’s asthma in South Korean bakers [38]. Higher levels of circulating MBL protein were related to baker’s asthma, as well as specific polymorphisms in *MBL2* gene.

Tsai et al. (2009) reported a genetic study on food allergies in American nuclear families [39]. They found that both genetics and environmental factors are critical in food allergies among the population under study. Using IgE-mediated food allergy as a genetic trait, they estimated that sensitization to food, including wheat, was controlled genetically with a heritability of 0.15–0.35 (Table 1). They did not study any specific type of gene polymorphisms.

In summary, there is growing evidence for a genetic basis underlying the development of both WDEIA and wheat allergy. However, the specific genetic polymorphisms underlying these two conditions appear to be different. Genetic polymorphisms associated with increased risk of developing WDEIA are as follows: IL-18, IL-4, and HLA class II DPB. In contrast, increased risk of developing wheat allergy sensitization or disease is associated with polymorphism of the following genes: TLR4, RBFOX1, IL-4 alpha receptor, and HLA class II DQ. The filaggrin gene’s loss-of-function mutation with relation to WDEIA is therefore controversial.

### 2.3. Role of Environment Factors in the Development of Wheat Allergy and Anaphylaxis

There are a limited number of studies examining the role of environmental factors in wheat allergy and anaphylaxis. The role of exposure to cats, gut microbiome diversity, Vitamin D deficiency, and the use of antacids have been studied as potential environmental factors associated with wheat allergy (Table 2).

In a Japanese study, exposure to cats during pregnancy and early infancy were studied. The researchers reported that such exposure to cats may prevent the development of wheat allergy. However, the duration of protection and whether such protection influences anaphylaxis are unknown [46].

The role of gut microbiome diversity in food allergy is a highly researched topic. However, there is only one study associated with wheat allergy. Savage et al. (2018) reported gut microbiome variation in general food allergy that included wheat allergic sensitization. They found a reduction in four genera of bacteria in subjects that had developed sensitization to food allergens, including wheat. However, the number of children with wheat sensitization was very low (*n* = 3–14), preventing the generalization of findings [47].

There is a significant deficiency of vitamin D in human populations living in colder areas of the world, such as North America. There is also growing evidence that Vitamin D deficiency may be an important factor in the development of allergic diseases in general [50]. However, there is only one study that reported the association of vitamin D with wheat allergy. Baek et al. (2014) studied the prevalence of sensitization to wheat among South Korean children with vitamin D deficiency and reported an increased risk of sensitization to wheat by four folds in this population [48].

Stomach acidity plays an important role in the breakdown of food allergens [51]. Therefore, the chronic usage of antacids such as (HIIR) blockers or proton pump inhibitors (PPI) may prevent the breakdown of allergens, thus facilitating allergic sensitization. There is one study associating antacids with sensitization to food allergens, including wheat [49] (Table 2).

We thoroughly reviewed the relationship between the environmental factors (farming environment, drinking unpasteurized milk, exposure to bacterial endotoxins, and contact with livestock at fetal stage and early childhood) and the development of airways allergies, food allergies (including wheat allergies), asthma, and atopic dermatitis. There are several reports in the literature suggesting significant relationships between these environmental factors and the development of airways allergies, asthma, and atopic dermatitis. Notably, these studies did not include food allergies or wheat allergies in their research [52,53]. Furthermore, in another independent study, no relationship was found between farming factors and food allergies, including wheat allergies [54]. Thus, the implications of hygiene hypothesis on wheat allergies remain to be fully investigated in the future (Table 2).

In summary, very limited information is available on the protective versus promotive effects of environmental factors in the development of wheat allergy. There is therefore ample opportunity to investigate the protective versus promotive role of environmental factors in the development of wheat allergy and anaphylaxis using validated animal models for the effects of environmental factors in wheat allergy development.

## 3. Mechanisms of Anaphylaxis in Wheat Allergy

In this section, we review the current understanding of the mechanisms involved in systemic anaphylaxis associated with wheat allergy. In the first part, we discuss this problem in humans and information from mouse models of classical anaphylaxis caused by wheat exposure. In the second part, we discuss wheat-dependent cofactor-induced/enhanced systemic anaphylaxis in humans and animal models.

### 3.1. Mechanism of Classical Anaphylaxis in Wheat Allergy

**Figure 1:**
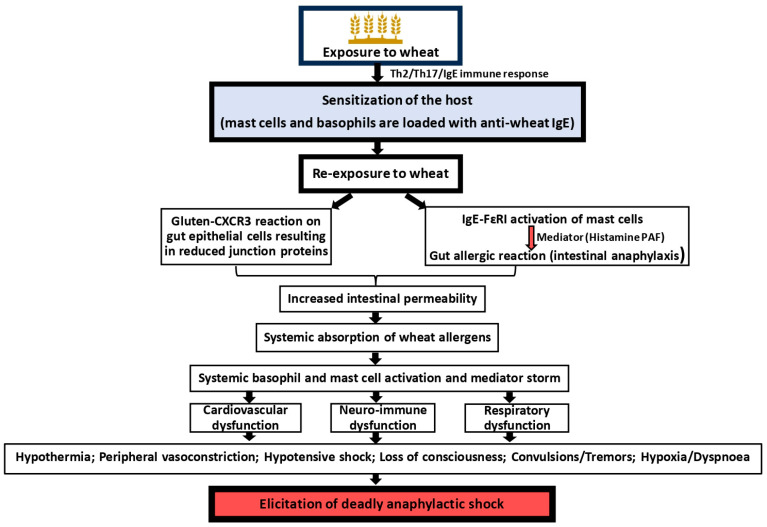
Mechanisms of elicitation of classical wheat-induced anaphylaxis. Exposure of genetically susceptible host to wheat proteins via oral, skin, eyes, and airways can activate the immune system to produce wheat-specific IgE antibodies. Tissue mast cells and blood basophils bind to IgE via a high-affinity receptor (FcεRI), resulting in sensitization. Subsequent exposure to wheat elicits potentially deadly systemic anaphylaxis via the pathways illustrated in the figure [56,57,58].

An overview of the current status of scientific knowledge on the classical wheat-induced anaphylaxis is illustrated in Figure 1. The first requirement for systemic anaphylaxis is the prior sensitization of the host to wheat-derived proteins. Both gluten proteins (gliadins and glutenin) and non-gluten proteins (salt-soluble proteins such as alpha amylase inhibitor) have been identified as sensitization-causing allergens. The mechanism of sensitization to wheat proteins is thought to occur as follows: The exposure of a genetically susceptible host to wheat allergens in the context of yet-to-be-identified abnormal environmental conditions results in immune responses characterized by T helper (Th)-2/Th17 dominance. The B lymphocytes specific to wheat allergens, under the influence of Th2/Th17 lymphocytes, start producing the IgE class of antibodies. The allergen-specific IgE antibodies then bind to FcεRI present on mast cells in the tissues and basophils in the blood. When the host reaches this stage of immune response, they are considered as sensitized to wheat allergens. In humans as well as animals, such sensitization is determined by measuring the allergen-specific IgE antibodies present in the blood using immune assays. In humans, clinical sensitization is established by performing a skin-prick test by administering a small quantity of allergens intradermally. A positive reaction occurs within a few minutes by the presentation of a wheal and flare reaction that is quantified by measuring the diameter of the reaction at 20–30 min. A positive control injection with histamine is commonly used to compare the reactions [55].

Re-exposure of a sensitized host to the same wheat allergen results in the clinical expression of allergic symptoms that can include local intestinal reactions resulting in vomiting and diarrhea; allergic reactions are also usually manifested by skin rashes. If the sensitized host is re-exposed to the allergen via the eyes and nose (as happens in baking/wheat flour industry workers), the allergic reactions are manifested in the respective organs as allergic conjunctivitis and allergic rhinitis. In severe cases, asthma attacks are known as baker’s asthma. If the reaction is restricted to this type of symptoms, it is not considered as systemic anaphylaxis. However, if the allergic reaction expands to other body systems besides the gastro-intestinal tract upon consumption of wheat-containing foods, it is regarded as systemic anaphylaxis. For example, simultaneous reactions occurring in the gut as well as the skin, airways, cardiovascular system, and neurological reactions upon consumption of wheat are all considered as systemic anaphylaxis. The mechanism of how a wheat allergic reaction progresses to become a systemic anaphylactic reaction is unclear at present. The current hypothesis is that wheat allergens that survive gastric-intestinal digestion leak into the blood and make their way to different organs of the body, causing allergic reactions systemically [59]. There is some evidence that gluten interactions with CXCR3 receptors on the gut epithelial cells can decrease the expression of zonulin proteins, resulting in increased permeability in the gut [60]. Additionally, the local allergic reaction in the intestine mediated by gut mast cell degranulation is amplified by unknown factors, resulting in extremely high histamine and PAF production. Intestinal permeability, as well as vascular permeability, will increase facilitating systemic absorption of wheat allergens. These processes are likely dependent on the dose of the allergen consumed, as well as the extent of IgE sensitization and the total number of IgE-sensitized mast cells in the gut. So, the higher the sensitization and mast cell density, the more intense the allergic reaction is expected to be in the intestine. Such a severe reaction is sometimes referred to as intestinal anaphylaxis [61].

Once the wheat allergens enter the blood stream from the gut, they bind to the IgE-sensitized basophils circulating in the blood, triggering a histamine reaction in the cardiovascular system. Furthermore, allergens reaching different vital organs via the blood can also cause IgE/FcεR1/mast cell degranulation in different organs, such as the heart, brain, and skin, and the airways. The resulting mediator immune storm is thought to contribute to the clinical symptoms of systemic anaphylaxis. The commonly reported symptoms of wheat-induced systemic anaphylaxis are hypothermia and tachycardia, followed by bradycardia, hypotensive shock, and loss of consciousness. The failure of cardiovascular as well as respiratory functions is attributed to deadly outcome from wheat-induced anaphylaxis. It is noteworthy that specific mediators of systemic anaphylaxis in different vital organs have not been well studied. The most commonly studied mediators of anaphylaxis include histamine, tryptase, and PAF [62].

### 3.2. Mechanism of Wheat-Dependent Exercise-Induced Anaphylaxis

**Figure 2:**
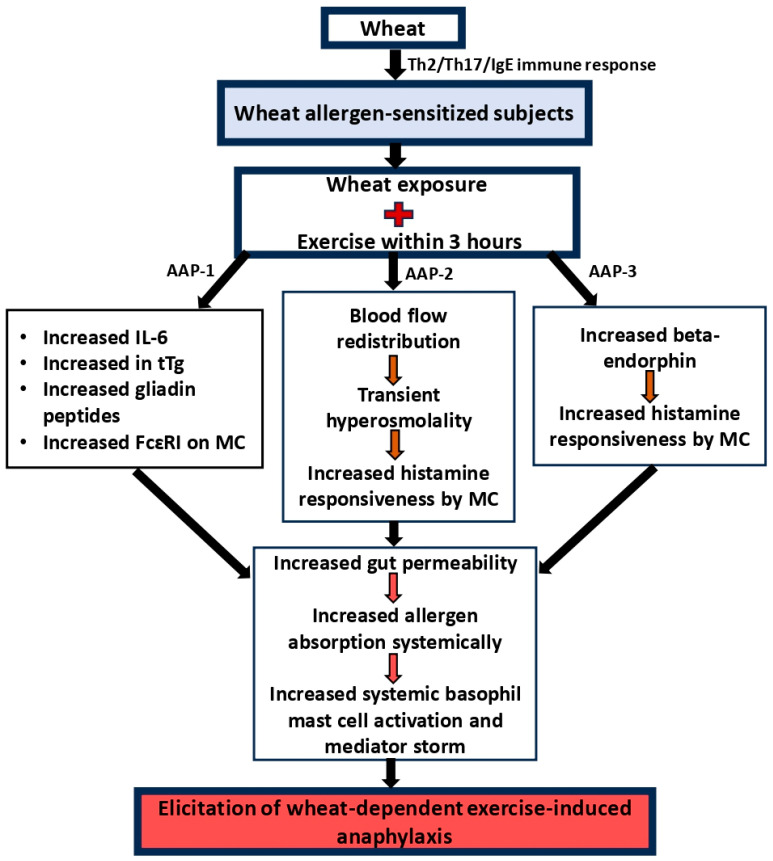
Mechanisms of elicitation of wheat-dependent exercise-induced anaphylaxis. Host sensitization to wheat proteins occurs in a similar fashion as described in Figure 1. However, subsequent exposure to wheat does not elicit systemic anaphylaxis unless the subject undergoes high-strain physical activity such as working out, marathon running, etc., that acts as the inducer of anaphylaxis rather than wheat per se. Although mechanisms underlying this condition are not completely clear, at least three anaphylaxis activation pathways (AAPs) have been proposed to explain the underlying pathophysiology, as illustrated in the figure [67].

There is sufficient evidence in the literature regarding the existence of food-dependent exercise-induced anaphylaxis in humans [63]. Wheat is among the implicated foods associated with this condition. Systemic anaphylaxis occurs in some wheat-allergic subjects upon consumption of wheat followed by high-strain physical activity, such as exercising, marathon running, etc. In these individuals, no such reaction is noted after consumption of wheat-containing food products. Mechanisms on how exercise induces anaphylaxis in wheat-sensitized subjects are reported as follows in Figure 2.

Sensitization to wheat allergy in these subjects is the requirement for this condition. The immunological mechanism, genetic predisposition, and the role of environmental factors associated with sensitization to wheat allergens appear to be like those individuals with classical systemic anaphylaxis, as discussed in the previous section. It is unclear as to how and why WDEIA patients can tolerate wheat when they do not perform high-strain physical activity. Upon exercise, three anaphylaxis activation pathways (AAPs) are identified, as discussed below.

AAP-1: Exercise has been shown to increase the expression of pro-inflammatory/allergic cytokine IL-6 [64]. Exercise has also been shown to increase the expression of tissue transglutaminase enzyme activity, leading to increased breakdown of gluten protein into small gliadin peptides that are capable of activating mast cells via FcεR1 signaling.

AAP-2: Exercise causes blood flow redistribution, resulting in transient hyper-osmolality [65]. Under these conditions, mast cells exhibit enhanced histamine production.

AAP-3: Exercise causes the release of beta endorphins [66]. Under the influence of these hormones, mast cells exhibit hyper activity, causing excessive histamine release.

One or more of the above AAPs results in the same consequence: exaggerated gut permeability leading to leakage of wheat allergens from the intestine into the systemic circulation. Bioactive allergenic peptides can activate basophils in the blood and mast cells in other organs, such as heart and lungs. Thus, it is hypothesized that WDEIA patients can tolerate wheat when they are not active but develop systemic anaphylaxis upon increased physical activity due to the combination of the above mechanisms.

Defining clearly the pathophysiological mechanisms of WDEIA is an ongoing challenge facing the field. Regarding the mechanisms underlying EIA, the European Academy of Allergy and Clinical Immunology has published a position statement previously [68]. According to these experts, the mechanisms proposed in the literature up until 2015 lacked validity. They also recommended that a global research network be formed to address this issue and facilitate improved diagnoses and treatment of EIA. Since then, scientific knowledge in this area has advanced [69].

Gabler et al. (2022) reported novel findings in WDEIA [70]. It is generally assumed that only gluten proteins (ω5 gliadins) cause WDEIA. They studied skin-prick test and basophil activation in 12 patients with WDEIA and 10 control patients. Based on the results, they concluded that non-gluten proteins with unidentified epitopes are relevant in WDEIA and suggested the role of nutritional anti-trypsin inhibitor (ATI) as a potential candidate explaining a novel hypothesis to be researched in the WDEIA mechanism.

Scherf et al. (2019) studied the mechanism of WDEIA with a focus on the absorption of gliadin in the gut [71]. They examined the influence of cofactors such as exercise (aerobic and anaerobic), acetyl salicylic acid, alcohol, and pantoprazole on absorption of gliadin, as measured by serum levels of gliadin up to 2 h after consumption. Interestingly, they found the cofactors do not influence the absorption of gliadin in healthy subjects. They suggested that, instead, patients with WDEIA may have a predisposition, such as damaged intestinal epithelial or hyperresponsive intestinal epithelial. Other possibilities suggested include cofactor-induced blood flow redistribution, increased tissue transglutaminase activity, plasma osmolality, and acidosis, leading to excess histamine release basophils and mast cells.

### 3.3. The Potential Role of Emerging Immune Mediators or Pathways That Have Not Been Extensively Covered in the Current Literature

Recent research has identified novel immune pathways in systemic anaphylaxis in peanut allergic subjects and in a mouse model of food allergy using peanut as a model food allergen.

Watson et al. (2017) studied acute allergic reactions to peanuts in severely allergic children (n = 19) upon double-blind oral challenges in controlled clinical conditions [15]. Their goal was to identify the key molecular drivers of severe acute allergic reactions to peanuts that may be similar to systemic anaphylaxis. They identified the following molecular drivers by conducting advanced gene profile analysis using blood samples collected at baseline and at 2 h and 4 h after oral peanut or placebo challenge: LTB4R, PADI4, IL1R2, PPP1R3D, KLHL2, and ECHDC3. These genes were identified as drivers of severe reactions associated with changes in neutrophil, naïve CD4^+^ T cell, and macrophage. Thus, in contrast to the current paradigm that IgE, IgE^+^ B cells, mast cells, and the basophils pathway are critical players in severe allergic reactions, this study proposes a new pathway involving phagocytes and T cells as drivers of severe reactions in peanut allergy. These cells, genes, and pathways may be targeted for severe peanut allergy therapy. Although they used peanut allergic subjects in this study, the results may be applicable to other types of severe food allergic reactions, including wheat allergy, that need to be confirmed. In this study, due to ethical reasons, anaphylaxis was not deliberately induced. However, the reactions were severe enough to treat with epinephrine; therefore, the molecular drivers and pathways of severe allergic reactions may reflect key mechanisms leading to systemic anaphylaxis if untreated with epinephrine.

Using a mouse model, Bao et al. (2023) reported for the first time the involvement of the central nervous system in causing life-threatening anaphylactic shock caused by food proteins [14]. This study clearly demonstrated how mediators released by the mast cell upon allergen activation engage neurons and the brain to cause life-threatening anaphylactic shock. Hypothermic shock response as measured by a drop in body (rectal) temperature is a widely used objective measure of systemic anaphylaxis in rodent models of food allergy and other types of allergic reactions. It is generally assumed that it is caused by IgE/mast cell interaction. In this paper, authors identified the involvement of the body’s thermoregulatory neural circuits in hypothermic shock reaction. They identified mast cell-derived chymase as an activator of TRPV1^+^-sensitive neurons via protease-activated receptor-1. Because of this signal, the regulatory neural network stops brown adipose tissue-mediated thermal regulation, resulting in hypothermic reaction. This was an unexpected pathway in systemic anaphylaxis. Although this study used trinitrophenol (TNP)-specific IgE and TNP-ova albumin protein as a model allergen, these bindings may be relevant for wheat anaphylaxis, although this needs to be confirmed. Thus, a novel pathway and therapeutic target (chymase, mast cell chymase, and TRPV1) for anaphylaxis have been identified for further research.

### 3.4. Mechanism of Other Cofactor-Induced Anaphylaxis

It is reported that some wheat-allergic subject drugs, such as NSAIDs, alcohol, and infections can act as cofactors, resulting in a loss of tolerance to wheat, expressed as systemic anaphylaxis. The specific mechanisms of these conditions are little known. It is hypothesized that NSAIDs bind to membrane phospholipids and cause mitochondrial dysfunction, leading to gut damage [72]. Such loss of tissues can facilitate allergen absorption into the systemic compartment. Furthermore, NSAIDs can increase calcium channel activity and lipid-derived mediators of anaphylaxis that can increase mast cell responses.

Mechanisms on how alcohol and infections can act as cofactors for triggering systemic anaphylaxis are not well studied. Acetaldehyde produced upon metabolism of alcohol can act as a non-specific activator of mast cells [73].

Some infections, such as bacterial and viral infections, can act via innate immune receptors (e.g., TLRs) via the PAMP/PRR pathway that can release pro-inflammatory mediators, thus facilitating the absorption of allergens, subsequently triggering systemic anaphylaxis [74,75].

## 4. Utility of Rodent Models in Elucidating Mechanisms of Anaphylaxis in Wheat Allergy

### 4.1. Rodent Models of Classical Anaphylaxis in Wheat Allergy

#### 4.1.1. Adjuvant-Based Models

There are several rodent models reported in the literature to study wheat-induced anaphylaxis in wheat-sensitized animals using adjuvants such as alum, salicylic acid, and detergents. Most rodent models have used adjuvants (alum, detergent, etc.,) to elicit robust sensitization to wheat proteins (Table 3). Although the role of detergents in causing sensitization to gluten in the context of cosmetic exposure is plausible, alum adjuvant is not expected to be involved in human sensitization to wheat proteins. The use of adjuvants in rodent models helps in creating robust readouts of sensitization (for example, high levels of wheat-specific IgE antibodies) very quickly by activating inflammatory conditions at the site of injection of a mixture of wheat allergen plus adjuvant. Although, technically, it is easy to do this, the underlying mechanism of sensitization for anaphylaxis is different compared to the sensitization method that does not involve adjuvants [58]. Previously, researchers have expressed concerns about using adjuvant-based models for determining the intrinsic allergenicity of proteins because this approach increases sensitivity at the loss of specificity of the reactions [76]. Therefore, some researchers have proposed that adjuvant-free methods are ideal for establishing the intrinsic allergenicity of any protein, including conventional food proteins such as wheat allergens, as well as novel food proteins (for example, genetically engineered food proteins and chemically/physically altered food proteins) [77,78,79,80]. Thus, adjuvants have the potential to influence sensitization as well as the underlying mechanisms. Therefore, their use can influence anaphylactic reactions indirectly.

With the exception of one report that used guinea pigs, all others used mice or rats. The major characteristics of these models are reviewed below.

Tanaka et al. (2011) developed the first mouse model of wheat-induced anaphylaxis. Animals were sensitized to gliadin via an IP injection along with an alum adjuvant [81]. Oral challenge with a high dose of ω-5 gliadin was conducted to induce anaphylaxis. Sensitization was confirmed by SIgE measurements, and anaphylaxis was quantified by HSR. Sensitized mice developed mild reactions within 30 mins post-challenge. The strengths of the model are as follows: (i) the measurement of SIgE and (ii) the demonstration of appearance of allergens in the portal blood after oral challenge at 1 h. The limitations of the study are as follows: (i) mediators were not studied and (ii) non-gliadins were not studied.

Adachi et al. (2012) reported a transdermal sensitization mouse model using the tape stripping method followed by application of gluten + SDS detergent [82]. Anaphylaxis was elicited by IP challenge with gluten, and sensitization was measured using SIgE and SIgG1. It was noted that sensitized mice developed severe HSR (3 °C drop in rectal temperature at 30 min), as well as clinical symptom scores (3). The strengths of this study include the following: (i) the measurement of SIgE and IgG1; (ii) histamine measurement; and (iii) the study of spleen TH1 and TH2 cytokine. The limitations of the model are as follows: (i) repeated tape stripping (10 times) of the top layer of skin raises animal well-fare issues; (ii) an oral challenge was not conducted; and (iii) mMCP-1 was not studied.

Jin et al. (2017) reported a mouse model of anaphylaxis using IP sensitization with SSP and alum [85]. Disease elicitation was conducted by IP challenge with SSP. Mice exhibited modest clinical reaction of anaphylaxis, as evidenced by HSR (3 °C). The strengths of this model are as follows: (i) the measurement of SIgE, TIgE, mMCP-1, and spleen biomarkers. The limitations of this study include the following: (i) there was no study of glutens; (ii) there was a lack clinical symptoms scoring; and (iii) histamine measurements were not carried out.

Fu et al. (2022) reported a mouse model of anaphylaxis using enzyme digested in gluten [87]. Mice were sensitized by IP injection of protein plus alum adjuvant. Disease was elicited by IP challenge with gluten through IP route. They reported modest anaphylaxis, as evidenced by HSR (2.5–3 °C drop) and clinical symptoms score (2–3). The strengths of the study include the following: (i) the SIgE measurement; (ii) the Th1 and Th2 cytokine measurement; and (iii) the measurement of mast cell numbers in the intestine. The limitations of the study include the following: (i) the lack of measurement of histamine and mMCP-1.

Kohno et al. (2016) reported a guinea pig model of anaphylaxis using intragastric sensitization of gluten with salicylic acid [83]. Disease elicitation was conducted by IP challenge with gliadin. They reported severe anaphylaxis (4–6 clinical symptom score). The strengths of the model include the following: (i) this is the first guinea pig model of wheat-induced anaphylaxis and (ii) oral sensitization was measured. The limitations of the model include the following: (i) no antibodies were studied and (ii) mediators were not studied. Therefore, the immune basis for the observed clinical symptoms of anaphylaxis is unclear.

Yamada et al. (2019) reported the first rat (male) model of gliadin/gluten-induced anaphylaxis [85]. The rats were sensitized by IP injection with gliadin and alum. Disease elicitation was conducted by IV challenge with ω-5 gliadin/gluten. They reported very mild HSR (0.4 °C in 30 min). The strengths of this model include the following: (i) this was the first rat model of ω-5-induced anaphylaxis. The limitations of the study include the following: (i) no antibodies were studied and (ii) mediators were not studied. Thus, the immune basis of the observed mild HSR is unclear.

Yamada et al. (2022) reported a rat model of ω-5 gliadin/gluten-induced anaphylaxis using female rats [86]. The animals were sensitized by SC with proteins and alum adjuvant, and disease elicitation was carried out by IV challenge with the respective proteins. They reported very mild anaphylaxis evidenced by HSR (approximately 0.8–1.4 °C). The strengths of the model are as follows: (i) measurement of SIgE and SIgG1 was performed and (ii) this was the first animal model to use the SC route for sensitization and IV routes for challenge. The limitations of this model include the following: (i) mediators were not studied and (ii) non-gluten proteins were not studied.

In summary, most adjuvant-based models have used mice, followed by rats. The mouse models produced anaphylaxis reactions varying from mild to severe. It was observed that rat models produced very mild reactions. Additionally, only one guinea pig model reported severe reactions. The mechanisms of anaphylaxis were studied in mice, to a limited extent in rats, and not at all in guinea pigs. Thus, there is a critical need to advance the development of improved models examining mechanisms of anaphylaxis in more detail. For example, whether the use of an adjuvant itself contributes to increasing the ability of wheat proteins to cause anaphylaxis remains to be addressed.

#### 4.1.2. Adjuvant-Free Models

There are three studies reported to develop adjuvant-free mouse models of WIA using salt-soluble protein, alcohol-soluble gliadin, and acid-soluble glutenin (Table 4).

Gao et al. (2022, 2023) used a transdermal exposure (TDE) method to induce sensitization to SSP without using adjuvants such as alum, salicylic acids, or detergents [78,88]. Anaphylaxis was elicited by oral challenge with SSP. They reported severe anaphylaxis, as evidenced by HSR (3.5 °C, 30 min). The strengths of the model include the following: (i) it achieved skin exposure without damaging the skin, such as through the tape stripping used in other models; (ii) it used the oral route for disease elicitation; (iii) it studied IgE and IgG1 antibodies; (iv) it studied mMCP-1; and (v) it studied spleen biomarkers. The limitations include of the model include the following: (i) they did not study histamine and (ii) IP challenges were not carried out to induce HSR.

Jorgensen et al. (2023) reported the first adjuvant-free mouse model of anaphylaxis induced by gliadin [80]. Sensitization was conducted by TDE with gliadin. Disease was elicited by IP challenge with gliadin. They reported a life-threatening dramatic reaction of anaphylaxis, as evidenced by HSR (8 °C in 30 min). The strengths of this model include the following: (i) it was the first model to develop life-threatening anaphylaxis reactions and (ii) it studied IgE, mMCP-1, and spleen biomarkers. The limitations of the study include the following: (i) it did not report histamine responses.

Jorgensen et al. (2023) reported the first adjuvant-free mouse model of glutenin-induced anaphylaxis [79]. Mice were sensitized by TDE with glutenin. Disease was elicited by IP challenge with glutenin. They reported life-threatening anaphylaxis, as evidenced by HSR (8 °C, 30 min) and a clinical symptom score of 4. The strengths of this model include the following: (i) it was the first adjuvant-free animal model of glutenin-induced life-threatening anaphylaxis and (ii) it studied IgE, mMCP-1, and spleen biomarkers. The limitations of the study are as follows: (i) it did not report histamine response and (ii) it did not report oral challenge.

In summary, adjuvant-free skin sensitization mouse models have been developed for all three types of wheat allergen-induced systemic anaphylaxis. These models provide a unique opportunity to dissect the mechanisms of severe and life-threatening anaphylaxis induced by wheat allergens. Furthermore, they also provide the opportunity to determine the impact of food processing on altering the intrinsic capacity of wheat allergens to elicit anaphylaxis.

### 4.2. Rodent Models of WDEIA

There are three papers published using mice (2) and guinea pigs (1) on this topic (Table 5). The first model was based on using mice [89]. In this model, the authors used SSP, gliadin, and glutenin with alum to sensitize by using IP injection. Later, mice were orally challenged with protein and subjected to exercise, with measurement of time used for exhaustion. Sensitized mice exhibited SIgE for gliadin and glutenin but not for salt-soluble protein. Gliadin and glutenin-sensitized mice were exhausted by about 30 and 50 mins, respectively. SSP-sensitized mice did not significantly differ from the control—unsensitized mice. The strengths of the study were as follows: (i) it used three types of proteins and (ii) it was the first published animal model study on WDEIA. The limitations of the study are as follows: (i) it did not study clinical symptom scores and HSR; (ii) there are missing controls for V/P and P/P groups; and (iii) it did not study mediators.

Tanaka et al. (2011) reported a mouse model of WDEIA sensitization, which was conducted using gliadin and alum adjuvant via intraperitoneal injection [90]. Mice were later orally challenged with gliadin and subjected to exercise for 30 min. Mice developed mild anaphylaxis. Upon treadmill exercise, sensitized mice were exhausted by 3 h as opposed to control mice, which were running for up to 9 h. The strengths of the study are as follows: (i) measurement of HSR and (ii) measurement of SIgE. The limitations of this study are as follows: (i) mediators such as histamine were not measured and (ii) control groups were missing for v/p and p/p without exercise group. Therefore, it is unclear if the model is truly WDEIA or simply WIA.

Kohno et al. (2016) developed a guinea pig model using gluten and salicylic acid as an adjuvant [83]. Sensitization was conducted by oral route. Disease elicitation was carried out after oral gluten challenge plus exercise for 30 min. Clinical symptoms scores were recorded; the studied reported mild symptoms upon exercise in sensitized guinea pigs.

In summary, there are limited efforts to develop rodent models of WDEIA. The reported models show mild symptoms of anaphylaxis. However, the absence of additional control groups prevents confirmation of the exercise dependency of observed anaphylaxis in these models. Future studies are required to further develop and validate robust rodent models of WDEIA by including quantifiable readouts of anaphylaxis and analysis of mediators of anaphylaxis, such as histamine measurements.

### 4.3. Lessons Learnt from the Rodent Models and Potential Utility to Advance the Field

Extensive research using rodent models of wheat allergy-associated anaphylaxis has advanced the science with the following critical knowledge and opportunities for future research agendas:

1. All three species of laboratory rodents (rats, guinea pigs, and mice) can be used to develop models simulating the two critical aspects of human disease—namely, sensitization, as demonstrated by induction of wheat-specific IgE antibodies, and elicitation of systemic anaphylaxis, as demonstrated by clinical disease and/or disease markers, such as hypothermic response.
2. Similar to humans, both gluten (gliadin and glutenin), as well as non-gluten proteins, elicit sensitization and anaphylaxis in rodents.
3. In the reported rodent models, the symptoms of anaphylaxis vary broadly from mild to moderate, severe, and life-threatening reactions; this spectrum of variation is also noted in humans.
4. In rodent models, sensitization is typically quantified by measuring wheat-specific IgE antibodies; there are no reports of developing skin testing in rodents, in contrast to the skin-prick test commonly carried out in humans to determine sensitization. However, in dog models of wheat food allergy, such tests are routinely performed [91]. Therefore, it may be possible to develop such a test in rodents in the future.
5. In rodent models, identified immune mediators associated with anaphylaxis include not only a selected set of Th2/Th17 cytokines and chemokines but also histamine, PAF, and mMCP-1. There is ample scope to expand the mediator analysis to include novel targets for potential diagnosis and therapy.
6. There are two mouse models and one guinea pig model of WDEIA. In a mouse model, it was demonstrated that exercise leads to lesion formation in the intestine associated with increased gut permeability and leakage of glutenin allergen into portal circulation and appearance in the liver. However, in these studies, appropriate controls were not used. Therefore, it remains to be clarified whether leakage of allergens to the liver from the gut is caused by exercise or whether such leakage happens in classical wheat-induced anaphylaxis. The guinea pig mouse model provides another useful model to study mechanisms of WDEIA, for which, currently, there is very limited information in the literature [84].
7. There is strong direct evidence from rodent models that exposure to wheat proteins (both glutens and non-glutens) via undamaged skin can clinically sensitize the host to subsequent life-threatening systemic anaphylaxis caused by wheat proteins. These findings have further bolstered the case for wheat anaphylaxis as an occupational public health issue in the food industry (e.g., baking) where such exposures must be closely monitored, prevented, and managed.
8. There are no rodent models reported for wheat-dependent alcohol, drug, or infection-induced systemic anaphylaxis at present; clearly, rodent models are needed in this area.
9. Rodent models provide ample opportunity to elucidate the role of genetic and environmental factors in determining anaphylaxis in wheat allergy; however, they have not been explored so far—they therefore constitute areas for further research.
10. There is growing evidence that the food and industrial processing of wheat proteins can influence its allergenic properties; therefore, rodent models can be employed to determine the impact of processing technology on the anaphylaxis-eliciting properties of wheat proteins.
11. There is growing interest in using rodent models to test novel genetically modified wheats for food safety assessment; currently, this has been carried out using rats and guinea pigs, but mouse models offer improved opportunities for this application [8].
12. Currently, there are extensive efforts in the area of biomarker research to advance disease diagnosis, prevention, and management of anaphylaxis in general [92,93,94,95]; rodent models of wheat anaphylaxis are expected to be critical tools to advance this area of research.

### 4.4. Limitations of Rodent Models and Challenges in Translating Findings from Rodent Models to Human Clinical Settings

There are several limitations that must be considered when translating findings from rodent models of anaphylaxis to human clinical settings, including the following:

1. In humans, specific mechanisms of sensitization to wheat are thought to occur upon oral ingestion of wheat-containing foods, although wheat dust inhaled in bakery settings and skin exposure to gluten via cosmetics (soaps, detergents, shampoos, etc.) is also reported [26,30,31,37,38,96]. In contrast, rodent models generally use sensitization methods that are artificial (for example, IP injections) (Table 3, Table 4 and Table 5).
2. In humans, sensitization to wheat proteins occurs after exposure to a complex mixture of proteins as they exist in the food matrix. In contrast, purified wheat proteins [glutens (gliadins, glutenin) and non-glutens (albumin and globulins)] are used in most rodent models (Table 3, Table 4 and Table 5).
3. Most rodent models have used adjuvants such as alum and detergents to elicit detectable sensitization to wheat proteins (Table 3, Table 4 and Table 5); although the role of detergents in causing sensitization to gluten in the context of cosmetic exposure is plausible, alum adjuvant is not expected to be involved in human sensitization to wheat proteins [31,35,82].
4. Human wheat anaphylaxis is reported after oral exposure to wheat-containing foods [3,13]. In contrast, in rodent models, except for studies conducted by Gao et al. (2022, 2023), Tanaka et al. (2011), Jorgensen et al. (2023), Kohno et al. (2016), and Kozai et al. (2006), where oral wheat protein challenges were carried out to elicit systemic anaphylaxis, all other studies used intraperitoneal or intravenous challenge to elicit anaphylaxis (Table 3, Table 4 and Table 5) [78,80,81,83,88,89]. Unlike in humans, where IgE primarily causes wheat anaphylaxis upon oral exposure to wheat allergens, in mouse models, anaphylaxis upon IP challenge with wheat allergens involves both IgE- and IgG1-mediated activation mechanisms [17].
5. In humans, anaphylaxis is generally reported after exposure to wheat present in cooked food [13]. In contrast, all rodent models have used raw wheat proteins for eliciting anaphylaxis and inducing sensitization (Table 3, Table 4 and Table 5).
6. All rodent models used inbred strains of animals that are expected to be genetically identical for each type of strain. Therefore, results from such studies must be interpreted carefully for translation to humans, where the population is outbred in nature. There is ample opportunity to develop outbred rodent models to simulate human wheat anaphylaxis. Such efforts are already in place for other human diseases, including asthma, obesity, diabetes, and cardiovascular diseases [97,98,99,100].

### 4.5. Current Efforts and Future Directions to Refine the Rodent Models to Better Mimic the Full Spectrum of Human Anaphylactic Reactions and the Influence of Cofactors

Classical systemic anaphylaxis to wheat in humans is a complex immune-mediated reaction involving interaction between various wheat proteins (a mixture of gliadins, glutenin, albumins, and globulins in specific ratios as a component of the food matrix) and protein-specific IgE antibodies present on mast cells and basophils [71,101]. Keeping this in mind, rodent models require further refinement to simulate human exposure conditions. Currently existing rodent models show variability and in full spectrum of anaphylaxis (ranging from very mild to mild, moderate, severe, and life-threatening reactions). However, dose responses in animal models have not been determined completely. For example, it is possible to determine the lowest observable adverse effect levels (LOAELs) and no observable adverse effect levels (NOAELs) in rodent models. Then, the validity of the rodent response in rodent models needs to be compared to human LOAELs and NOAELs.

Cofactor-dependent (exercise, alcohol, infections, drugs, etc.) wheat anaphylaxis in humans has been well established, with incompletely understood mechanisms [31,32,34,64,66,67,68,70,96,102,103,104,105,106,107] (Figure 2). This is the area where very limited and less optimal work has been carried out in rodent models, with reports only on WDEIA. Clearly there is ample opportunity to improve rodent models of WDEIA as well as to create novel models of alcohol-, infection-, and drug-dependent wheat anaphylaxis models in future. Such models will be valuable both for basic work on mechanisms and for translational work involving novel therapeutic development, as well as for vaccine development for wheat anaphylaxis in humans.

## 5. Conclusions and Future Directions

Wheat allergy associated with anaphylaxis is a major food safety problem at the global level. All four groups of wheat allergens (gliadins, glutenin, albumin, and globulin) exhibit intrinsic allergenicity and anaphylaxis-eliciting properties. Wheat-associated anaphylaxis is a complex clinical phenotype. Current knowledge identifies two broad groups of wheat anaphylaxis—namely, classical wheat-induced anaphylaxis and wheat-dependent exercise-induced anaphylaxis. However, additional cofactors such as alcohol, drugs, and infections can also precipitate anaphylaxis in wheat-allergic subjects who can otherwise tolerate wheat consumption. There is a growing critical need discussed in the literature to advance the mechanisms underlying life-threatening versus non-life-threatening allergic reactions to wheat. The identification of specific biomarkers to distinguish various types of anaphylaxes in wheat allergies is a major research need at present. Several rodent models of wheat-induced anaphylaxis and WDEIA with their own strengths and limitations are described in the literature. These models provide unique opportunities to study mechanisms and biomarker discovery in this field. This research has identified the complexity associated with wheat-induced anaphylaxis in humans as well as rodent models. We have identified specific gaps and opportunities for future research to enable the development of improved methods for prevention, treatment, and management of wheat-induced anaphylaxis. Overall, this study represents a comprehensive updated critical resource for the development of future research agendas in wheat-allergy-associated anaphylaxis using rodent models as very attractive pre-clinical tools for this endeavor.

## Figures and Tables

**Table 2:** Environmental factors in wheat allergy.

Environmental Factors	Evidence from the Study	References
Exposure to cats	Exposure to cats during pregnancy reduced the risk of wheat allergy in children until the age of 3 years (aORs [95% CIs] 0.54 [0.34–0.85]); Exposure to cats during early infancy reduced the risk of wheat allergy until the age of 3 years (0.63 [0.42–0.92]); Japanese study	[46]
Gut microbiome	food (milk, egg, peanut, soy, wheat, and walnut) sensitization in adults was associated with reduction in the following genera: *Haemophilus*, *Dialister*, *Dorea*, and *Clostridium* sensitization (*n* = 85 total food sensitized, wheat sensitization *n* = 33); Food (including wheat) allergy in children was associated with a reduction in the following genera *Citrobacter*, *Oscillospira*, *Lactococcus*, and *Dorea*; wheat allergic children (*n* = 3) among food allergic subjects (*n* = 14); United States study, adults, pregnant women, infants.	[47]
Vitamin D deficiency	Vitamin D deficiency during childhood increased the risk of sensitization (specific IgE antibody) to wheat (OR 4.2; 95% CI 1.1–15.8); South Korean study.	[48]
Use of antacids/antiulcer medications	Antacids (H2R blocker or PPI) treatment for 3 months increased sensitization (IgE) to food allergens, including wheat (Total *n* = 152, Hungarian study, adults)	[49]

**Table 3:** Adjuvant-based rodent models of wheat-induced anaphylaxis.

Model	Anaphylaxis Severity	Mechanism	Suggestions for Improvement
B10 female mice and ICR mice; Sensitization: IP injection of gliadin with alum adjuvant. Elicitation: oral high dose gliadin, high dose ω5-gliadin for 30 min to induce WIA [81]	Mild HSR (<2 °C drop in temperature in 30 min).	SIgE, elevation of proteins in the portal blood after oral challenge at 1 h	Study cytokines, histamine, and other mediators
Female BALB/c mice; Sensitization: TDE (tape stripping); gluten + adjuvant Elicitation: IP challenge, gluten to induce HSR * [82]	Severe; HSR: (more than 3 °C, 30 min); anaphylactic score: 3 vs. 0 in control;	Histamine, Th1/Th2 cytokines, IgE, IgG1	Study mMCP-1, chemokines, and other immune markers
Male Kud: Hartley guinea pigs; Sensitization: fasting 16 h + intragastric administration with salicylic acid and, 1 h later, gluten solution. Repeated for 9 days; Elicitation: IP injection of gliadin to elicit classical anaphylaxis reaction [83]	Classical systemic anaphylaxis (Severe: 4–6 in IP group)	Unknown	Study: (1) time to exhaustion; (2) antibodies: SIgE/SIgG1; (3) mediators (histamine, cytokines, etc.)
Female Balb/c mice; Sensitization: IP; SSP+ Alum. Elicitation: IP, SSP to elicit HSR [57,84]	Modest severity; HSR: (3 °C, 30 min;)	SIgE, TIgE, mMCP-1, spleen cytokine, chemokine, adhesion molecule.	Study histamine
Male Bn rats; Sensitization: IP, ω5-gliadin + Alum. Elicitation: IV, ω5-gliadin or gluten extract to elicit HSR [85]	Very mild; HSR: (0.4 °C, 30 min)	Unknown	Study antibodies, mediators (histamine, cytokines, etc.)
Female BN rat; Sensitization: TCI gluten ω5-gliadin + alum, SC. Elicitation: IV, TCI gluten or ω5-gliadin to elicit HSR [86]	Very mild; HSR: (approx. 0.8–1.4 °C, 30 min)	SIgE and SIgG1	Study mediators (histamine, cytokines, etc.)
Female Balb/c mice; Sensitization: IP, peptin + trypsin digested gluten + alum. Elicitation: IP, gluten to elicit HSR ** [87]	Modest Severity; HSR: (2.5–3 °C, 30–60 min); Clinical scores: 2–3 by 60 min	SIgE, increased mast cell number in duodenum. Reduced Th1 cytokine (spleen)	Study other cytokines, histamine, and other mediators

* Allergy scores were calculated as follows: 0 = no symptoms; 1 = pilar erecti, scratching, and rubbing around the nose and head; 2 = redness and puffiness reaction, diarrhea; 3 = wheezing, labored respiration, and cyanosis around the mouth and tail; 4 = death [82]. ** Allergy scores were calculated as follows: 0 = no symptoms; 1 = repetitive mouth/ear scratching and ear canal digging with hind legs; 2 = decreased activity, self-isolation, and puffiness around the eyes and/or mouth; 3 = periods of motionlessness for more than 1 min, lying prone on stomach, and decreased activity; 4 = no response to whisker stimuli and reduced or no response to probing; 5 = tremor, convulsion, and death [87]. Abbreviations used in the table: WIA—wheat-induced anaphylaxis; HSR—hypothermic shock response; SSP—salt-soluble protein; IP—intraperitoneal; SC—subcutaneous.

**Table 4:** Adjuvant-free rodent models of wheat-induced anaphylaxis.

Model	Anaphylaxis Severity	Mechanism	Suggestions for Improvement
Female Balb/c mice; sensitization: TDE, SSP; Challenge: Oral, SSP to elicit HSR [78,88]	Severe; HSR: (around 3.5°, 30 min)	TIgE, SIgE, SIgG1, mMCP-1, spleen biomarkers	Study histamine, IP challenge
Female Balb/c mice; Sensitization: TDE; Gliadin; Challenge: IP/oral Gliadin to elicit HSR * [80]	Life threatening; HSR: (8 °C, 30 min); Clinical symptoms scores 4	TIgE SIgE, mMCP-1, spleen biomarkers	Study histamine, oral challenge
Female Balb/c mice; Sensitization: TDE; Glutenin; Challenge: IP Glutenin to elicit HSR * [79]	Life threatening; HSR: (8 °C, 30 min); Clinical symptom score: 4 vs. 0 in control	TIgE SIgE, mMCP-1, spleen biomarkers	Study histamine, oral challenge

* Allergy scores were calculated as follows: 0 = no symptoms; 1 = scratching and rubbing around the nose and head; 2 = puffiness around the eyes and mouth, diarrhea, pilar erecti, reduced activity and/or decreased activity with a noted increase of respiratory rate; 3 = wheezing, labored respiration, and cyanosis around the tail and the mouth; 4 = no activity after prodding, tremors, and convulsions; 5 = death [79,80]; Abbreviations used in the table: HSR—hypothermic shock response; SSP—salt-soluble protein; IP—intraperitoneal.

**Table 5:** Rodent models of wheat-dependent exercise-induced anaphylaxis.

Model	Anaphylaxis Severity	Mechanism	Suggestions for Improvement
Female B10.A mice; Sensitization: IP, protein (salt-soluble protein, gliadin, and glutenin) + alum; Elicitation: Oral protein + treadmill to induce WDEIA [89]	Treadmill exhaustion time: gliadin and glutenin 35–50 min vs. control 150 min vs. control (*v*/*v*) 200 min	SIgE; Poor response to SSP and good response to gliadin and glutenin. Mucosal lesions in small intestine, leakage of proteins into blood and liver after challenge	Controls missing for V/P and P/P without exercise; Therefore, unclear if this model is truly WDEIA or just WIA; Study mediators
B10 female mice and ICR mice; Sensitization: IP injection of gliadin with alum adjuvant. Elicitation: oral gliadin + treadmill for 30 min to induce WDEIA [90]	Mild HSR (1.5 degrees drop in temperature in 30 min). Treadmill exhaustion test: the mice were exhausted by 3 h and remained so up to 9 h (revolutions stay <400 up to 9 h post-challenge vs. around 1000 in control) control—unsensitized mice orally challenged with vehicle (acetic acid 0.1 M).	SIgE	Controls missing for V/P and P/P without exercise; Therefore, unclear if this model is truly WDEIA or just WIA; Study cytokines, histamine, and other mediators
Male Kud: Hartley guinea pigs; Sensitization: fasting 16 h + intragastric administration with salicylic acid and, 1 h later, gluten solution. Repeated for 9 days; Elicitation: oral gluten + treadmill for 30 min to elicit WDEIA [83]	WDEIA clinical symptom scores * (Mild: 1–1.4 in oral + exercise group)	Unknown	Controls missing for V/P and P/P without exercise; Therefore, unclear if this model is truly WDEIA or just WIA; Study: (1) time to exhaustion: (2) antibodies: SIgE/SIgG1; (3) Mediators (histamine, cytokines, etc.)

* Allergy scores were calculated as follows: 0 = no symptoms; 1 = pilar erecti, scratching and rubbing around the nose and head; 2 = redness and puffiness reaction, diarrhea; 3 = wheezing, labored respiration, and cyanosis around the mouth and tail; 4 = death [84]. Abbreviations used in the table: WDEIA—wheat-dependent exercise-induced anaphylaxis; WIA—wheat-induced anaphylaxis; HSR—hypothermic shock response; SSP—salt soluble protein; IP—intraperitoneal; V/P—vehicle/protein; P/P—protein/protein.

## Data Availability

The original contributions presented in this study are included in the article. Further inquiries can be directed to the corresponding author.

## Abbreviations

WDEIA	Wheat-dependent exercise-induced anaphylaxis
WAO	Wheat allergy organization
TLR4	Toll-like receptor 4
LPS	Lipopolysaccharide
IL-4	Interleukin 4
IL-4R	Interleukin-4 receptor
HWP	Hydrolyzed wheat protein
HLA	Human leukocyte antigen
IL-18	Interleukin-18
MBL	Mannose-binding lectin
RBFOX1	RNA-binding fox-1 homolog 1
aOR	Adjusted odds ratio
PPI	Proton pump inhibitor
TNP-ova	Trinitrophenyl-ovalbumin
Th2, Th17	T helper2, T helper17
CXCR3	C-X-C motif chemokines receptor 3
PAF	Platelet-activating factor
AAPs	Anaphylaxis activation pathways
ATI	Anti-trypsin inhibitor
NSAIDs	Nonsteroidal anti-inflammatory drugs
PAMP	Pathogen-associated molecular pattern
PRR	Pattern recognition receptors
LOAELs	Lowest observable adverse effect levels
NOAELs	No observable adverse effect levels
WIA	Wheat-induced anaphylaxis
IP	Intraperitoneal
HSR	Hypothermic shock response
TDE	Transdermal exposure
mMCP-1	Murine mucosal mast cell protease-1
SSP	Salt-soluble protein
IV	Intravenous
SC	Subcutaneous
V/P	Vehicle application over the skin/protein injection IP
P/P	Protein application over the skin/protein injection IP
SIgE	Specific IgE antibodies
TIgE	Total IgE antibodies
